# Convex-hull voting method on a large data set

**DOI:** 10.1186/1471-2105-16-S15-P2

**Published:** 2015-10-23

**Authors:** Sally R Ellingson, Chi Wang, Radhakrishnan Nagarajan

**Affiliations:** 1 Division of Biomedical Informatics, College of Public Health, University of Kentucky, Lexington, KY 40536, USA; 2 Division of Cancer Biostatistics, College of Public Health, University of Kentucky, Lexington, KY 40536, USA; 3 Cancer Research Informatics Shared Resource Facility, Markey Cancer Center, Lexington, KY 40536, USA; 4 Biostatistics and Bioinformatics Shared Resource Facility, Markey Cancer Center, Lexington, KY 40536, USA

## Background

Genes work in concert as a system, not as independent entities, to mediate disease states. There has been considerable interest in understanding variations in molecular signatures between normal and disease states. The selective-voting convex-hull ensemble procedure accommodates molecular heterogeneity within and between groups and allows retrieval of sample-specific sets and investigation of variations in individual networks relevant to personalized medicine[1]. The work here describes using the convex-hull voting method on a large data set. Using parallelization techniques, we predict that we can execute the convex-hull voting algorithm on the University of Kentucky cluster (DLX) using a dataset much too large to run in a feasible time on a single machine.

## Materials and methods

**Figure 1:**
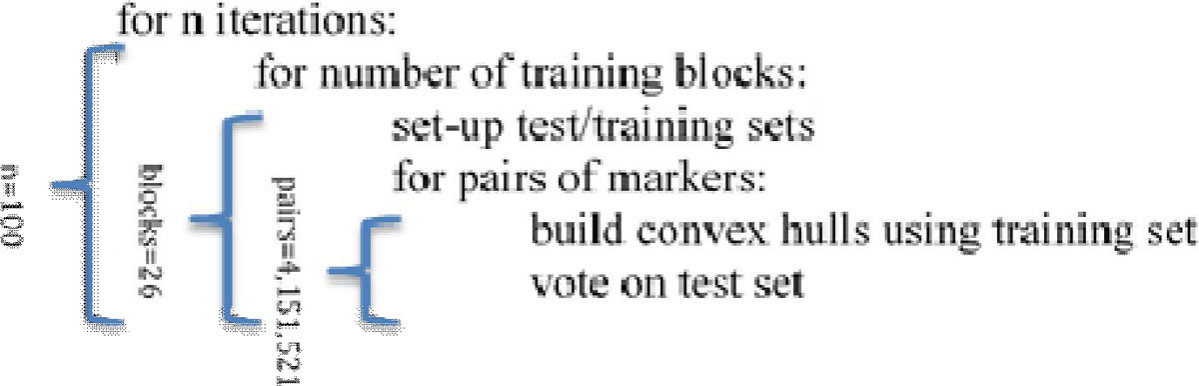
Ensemble convex-hull voting algorithm and levels of parallelization

Normalized RNA-seq data for 208 samples (104 matched normal/tumor pairs) from TCGA breast carcinoma data set were downloaded and analyzed by the edgeR package, which identified 2,882 differentially expressed genes with at least a 2-fold difference between tumor and normal samples and at 1% false discovery rate. The convex-hull voting method^1^ was applied to data from the differentially expressed genes. A general idea of the algorithm including levels of parallelism is given in Figure 1.

A parallel-for loop is used within the R code allowing multiple processors within a node to concurrently perform the voting calculations of different sample pairs within one iteration. Then multiple jobs are submitted to perform the randomized iterations. This turns a computationally intensive problem into a data intensive problem since each iteration produces just over 6 GBs of data.

## Results

The final runtime of one iteration of the large dataset was just under 34 hours and up to 32 iterations can run concurrently. The entire run of 100 iterations using this large data set took less than a week time.

## Conclusions

Future work will involve the parallelization of the entire computationally and data intensive steps in a way that reduces the complexity of job submission and scalability of the entire job. Computing paradigms such as Hadoop are being explored for this task.
